# Short Sleep Duration Measured by Smartwatch Is Associated With Elevated Resting Heart Rate and Reduced Nocturnal Oxygen Saturation: Insights From Heartbeat

**DOI:** 10.1111/jce.70358

**Published:** 2026-05-26

**Authors:** Alex El Darzi, Carlo El Khoury, Christian M. Massad, Mohammad Montaser Atasi, Michel Abou Khalil, Yara Menassa, Nadim Chaarani, Chanho Lim, Han Feng, Nassir F. Marrouche

**Affiliations:** ^1^ Tulane Research Innovation for Arrhythmia Discovery Tulane University School of Medicine New Orleans Louisiana USA; ^2^ Faculty of Medicine University of Balamand Balamand Koura Lebanon

**Keywords:** cardiovascular outcomes, maximum nocturnal oxygen saturation, oxygen saturation, resting heart rate, short sleep duration, wearable devices

## Abstract

**Introduction:**

Sleep duration has been shown to impact cardiovascular outcomes; however, the impact on wearable‐derived metrics such as oxygen saturation and resting heart rate remains underinvestigated. This study aims to determine whether average sleep duration of ≤ 6 h per night impacts resting heart rate (RHR) and maximum nocturnal oxygen saturation (MaxSpO₂).

**Methods:**

Using data from the HEARTBEAT study, sleep duration and maximum oxygen saturation during sleep were collected via the Samsung Galaxy Watch. Participants were stratified into two groups based on average sleep duration (≤ 6 h vs > 6 h per night) and propensity‐matched for baseline characteristics. The primary outcomes were RHR and MaxSpO₂. Statistical analysis was performed using R, version 4.5.1.

**Results:**

After propensity score matching, there were 168 patients in each group with well‐balanced baseline characteristics and comorbidities. Participants with average sleep duration ≤ 6 h demonstrated higher RHR compared to those with average sleep duration > 6 h (61.0 ± 5.8 bpm vs 57.7 ± 6.8 bpm; *p* < 0.001). Additionally, MaxSpO₂ was lower in the group with shorter sleep duration (95.1 ± 2.4% vs 96.2 ± 1.5%; *p* < 0.001).

**Conclusion:**

Sleeping ≤ 6 h per night is associated with higher resting heart rate and lower maximum nocturnal oxygen saturation.

## Graphical abstract

Short Sleep Duration and Wearable‐Derived Cardiovascular Metrics.

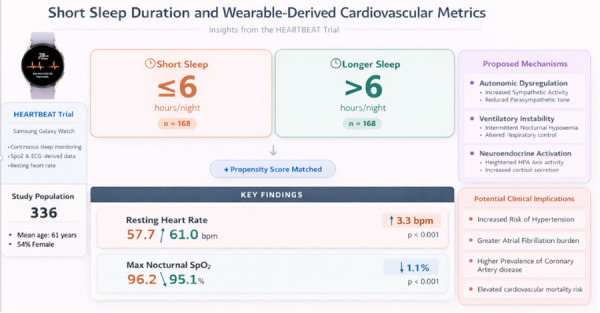

**Abbreviations**
CAD: Coronary artery disease
HEARTBEAT: Health Electronic Assessment of Risks and Trends Using Biometric Equipment and Technology
MaxSpO_2_
: Maximum blood oxygen saturation during sleep
OSA: Obstructive sleep apnea
PPG: Photoplethysmogram
RHR: resting heart rate

## 1 Introduction

Sleep duration plays a crucial role in cardiovascular health. Short sleep duration, defined as less than 6 h of sleep per night, has been associated with adverse cardiovascular outcomes including hypertension, coronary artery disease (CAD), arrhythmias, and mortality [1]. The mechanisms behind this include increased sympathetic nervous system activity, reduced parasympathetic modulation, systemic inflammation, oxidative stress, and impaired endothelial function [2, 3].

Resting heart rate (RHR) and nocturnal blood oxygen saturation (SpO₂) are two physiological parameters that reflect cardiovascular and respiratory health. RHR is a strong indicator of cardiovascular well‐being, with higher values predicting higher mortality rates and major adverse cardiovascular events (MACE) [4, 5]. Nocturnal oxygen desaturation may indicate underlying sleep disturbances which in turn are associated with poor cardiovascular outcomes. Slight reductions in SpO₂ have been associated with increased sympathetic tone, impaired vascular function, and increased long‐term cardiovascular risk [6].

With advancements in technology and improved patient cooperation, wearable devices such as smartwatches are now able to continuously monitor various biometric parameters [7, 8]. The Samsung Galaxy Watch, for example, measures heart rate, heart rate variability (HRV), SpO₂, ECG, photoplethysmogram (PPG), bioelectrical impedance analysis, and stress levels, among many other parameters. This enables physicians to better understand human physiology and pathophysiology through a biometric lens. Despite the increased clinical use of wearables, the direct impact of short sleep duration on wearable‐derived metrics such as RHR and SpO₂ has not been thoroughly investigated.

In this study, we used HEARTBEAT to evaluate whether sleep duration of more or less than 6 h, measured with the Samsung Galaxy watch, is associated with differences in RHR, and maximum blood oxygen saturation during sleep (MaxSpO₂).

## 2 Methods

### 2.1 Study Design

This analysis was conducted as part of the HEARTBEAT study, which is a prospective study designed to assess the value of wearable watch data in managing and predicting patients at risk of cardiovascular conditions. HEARTBEAT was approved by the Institutional Review Board (IRB) (NCT06753045), and all participants provided informed consent. Participants were recruited from diverse clinical settings to reflect a real‐world cardiovascular population. The study utilized a hybrid recruitment strategy, including clinical enrollment from cardiology and primary care clinics as well as remote and community‐based recruitment pathways. Adults aged 18 years or older with available sleep and wearable‐derived physiologic data were eligible for the present analysis. Participants with missing data such as sleep metrics or non‐adherence to wearing the watch were excluded from this study.

### 2.2 Study Procedures and Data Acquisition

At enrollment, Samsung smartwatches were provided to all participants at no cost, without requiring prior ownership. Participants were instructed to wear the smartwatch for at least 18 h per day, including overnight, and they received training on device use and study procedures. Automated reminders and a compliance‐monitoring system were used throughout follow‐up.

Sleep was determined using the Samsung Health algorithm, which integrates accelerometer‐derived movement with physiological signals from PPG (including heart rate and related features) to distinguish sleep from wakefulness, rather than relying on inactivity alone. Data was collected using the Samsung Galaxy atch, which provides continuous sleep monitoring and PPG data. Sleep duration was calculated nightly, in hours, averaged across all available nights up to the day of analysis. RHR was defined as the individual's average heart rate during rest, in beats per minute. MaxSpO₂ was defined as the highest oxygen saturation measured by PPG among all values recorded during the night. RHR and oxygen saturation were derived from PPG‐based smartwatch measurements. Maximum nocturnal SpO₂ was assessed during sleep, a period associated with reduced motion artifact and improved signal quality. For RHR, multiple PPG strips were reviewed to confirm the reliability of the algorithm‐derived measurements, and were found to be highly concordant with our own manual readings. For both RHR and SpO₂, the algorithms were applied only to clean PPG signals with minimal noise.

Baseline characteristics and clinical data were obtained from electronic health records and included: age, sex, body mass index (BMI), heart failure (HF), chronic kidney disease (CKD), hypertension, obstructive sleep apnea, CAD, and diabetes mellitus, as well as hospitalizations and emergency visits.

### 2.3 Group Stratification and Matching

Participants were stratified into two groups based on their average sleep duration: ≤ 6 h or > 6 h. The 6‐h threshold was chosen based on previous definitions of short sleep and its negative effects on cardiovascular health [9]. The primary outcomes of our study were RHR and MaxSpO₂.

To control for confounding variables, propensity score matching was performed. The propensity score model included age, sex, BMI, hypertension, HF, CKD, diabetes, CAD, and OSA. The balance of the variables after matching was assessed using a regression model, which showed no significant differences between sleep groups for demographic and clinical variables. These results demonstrate that both groups were balanced for baseline characteristics and comorbidities.

### 2.4 Statistical Analysis

Baseline characteristics before and after matching were compared. Continuous variables were compared using the Wilcoxon test. Categorical variables were compared using the Chi‐square test. The primary outcomes, RHR and MaxSpO₂, were compared between the matched short sleep group and the longer sleeping group using the Wilcoxon test. A two‐sided *p*‐value of < 0.05 was considered statistically significant. All analyses were performed using R statistical software, version 4.5.1.

Sleep duration was also analyzed as a continuous independent variable using a multivariable linear regression model to evaluate its association with maximum oxygen saturation (MaxSpO₂), HRV, and RHR, while adjusting for age, sex, BMI, and CHA₂DS₂‐VASc score. Beta coefficients (β), standard errors (SE), 95% confidence intervals (CI), and two‐sided p‐values were reported, with statistical significance defined as *p* < 0.05.

Hypoxia burden was also assessed, defined as the average proportion of hypoxic episodes per sleep cycle. A hypoxic event was defined as an oxygen saturation < 90%. For each sleep cycle, hypoxia burden was calculated as the number of episodes with oxygen saturation < 90% divided by the total number of recorded episodes, and this value was then averaged across sleep cycles. For categorical analysis, the association between sleep duration and hypoxia burden (defined as hypoxia burden ≥ 1%) was assessed using Fisher's exact test. Odds ratios (ORs) with 95% confidence intervals (CIs) were reported.

## 3 Results

### 3.1 Baseline Characteristics

The initial cohort comprised 434 participants. At baseline, 253 (58%) had an average sleep duration of ≤ 6 h per night, and 181 participants (42%) had an average of > 6 h per night. Prior to matching, the participants who slept ≤ 6 h had a higher proportion of comorbidities such as hypertension (*p* = 0.026) and diabetes (*p* = 0.021). Furthermore, participants had similar rates of beta blockers (*p* = 0.055) and antiarrhythmic drug use (*p* = 0.75) (Table 1). After propensity matching, 336 total participants remained, with 168 participants in each group. All measured baseline characteristics were well‐balanced and non‐significant between the two groups (Table 2). The matched cohorts had a mean age of 62.8 years, were 46.4% male and 53.6% female, and had similar distributions of BMI (*p* = 0.78), HF (*p* = 0.44), CKD (*p* = 0.86), diabetes (*p* = 0.64), CAD (*p* = 0.72), OSA (*p* = 0.88), hypertension (*p* = 0.91), hospitalization (*p* = 0.65), and emergency visits (*p* = 0.761).

**Table 1:** Baseline medication use stratified by sleep duration (≤ 6 h vs > 6 h) before propensity score matching.

Medications	≤ 6 h (*n* = 253)	> 6 h (*n* = 181)	P_value
BetaBlockers	124 (49)	71 (39.2)	0.0545
Statin	148 (58.5)	93 (51.4)	0.1697
Insulin	21 (8.3)	8 (4.4)	0.1611
Levothyroxine	24 (9.5)	18 (9.9)	1
AADClassI	5 (2)	5 (2.8)	0.7478
AADClassII	81 (32)	53 (29.3)	0.6153
AADClassIII	21 (8.3)	9 (5)	0.2478
AADClassIV	5 (2)	7 (3.9)	0.3746

**Table 2:** Baseline characteristics and comorbidities before and after propensity matching.

Variable	Before Matching			After Matching		
	≤ 6 h (*n* = 253)	> 6 h (*n* = 181)	P‐value	≤ 6 h (*n* = 168)	> 6 h (*n* = 168)	P‐value
Age, mean (SD), years	61.11 (12.40)	62.15 (14.49)	0.421	62.36 (12.40)	63.32 (13.76)	0.503
BMI, mean (SD)	31.75 (7.67)	30.28 (7.20)	0.05	30.49 (7.14)	30.28 (7.20)	0.781
Female, *n* (%)	121 (47.8)	97 (53.6)	0.277	88 (52.4)	92 (54.8)	0.743
Male, *n* (%)	132 (52.2)	84 (46.4)	0.277	80 (47.6)	76 (45.2)	0.743
HF, *n* (%)	48 (19.0)	21 (11.6)	0.053	27 (16.1)	21 (12.5)	0.436
CKD, *n* (%)	41 (16.2)	19 (10.5)	0.119	21 (12.5)	19 (11.3)	0.866
HTN, *n* (%)	173 (68.4)	104 (57.5)	0.026	105 (62.5)	103 (61.3)	0.911
OSA, *n* (%)	73 (28.9)	30 (16.6)	0.004	28 (16.7)	30 (17.9)	0.885
CAD, *n* (%)	83 (32.8)	54 (29.8)	0.581	50 (29.8)	54 (32.1)	0.723
DM, *n* (%)	101 (39.9)	52 (28.7)	0.021	57 (33.9)	52 (31.0)	0.641
Hospitalization, *n* (%)	17 (6.7)	9 (5.0)	0.582	12 (7.1)	9 (5.4)	0.652
Emergency Visits, *n* (%)	45 (17.8)	24 (13.3)	0.255	27 (16.1)	24 (14.3)	0.761

### 3.2 Resting Heart Rate and Sleep Duration

**Figure 1:**
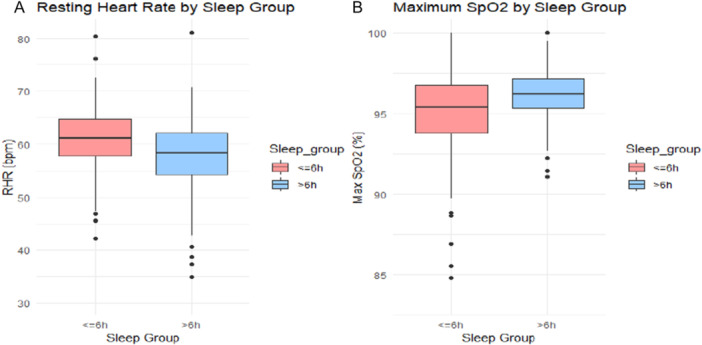
(A) Box plot showing significantly higher Resting Heart Rate (RHR) in the Short Sleep (≤ 6 h) group (61 bpm) compared to the longer sleeping group (> 6 h) (57.7 bpm), *p* < 0.001. (B) Box plot showing significantly lower maximum nocturnal oxygen saturation (MaxSpO_2_) in the Short Sleep group (95.1%) compared to the longer sleeping group (96.2%), *p* < 0.001.

Participants who slept on average ≤ 6 h showed significantly higher RHR compared to those who slept more than 6 h. The shorter sleep group had an RHR of 61.0 ± 5.8 bpm in comparison to the longer sleeping group who had an RHR of 57.7 ± 6.8 bpm (mean difference +3.3 bpm, *p* < 0.001) (Figure 1).

### 3.3 MaxSpO_2_ and Sleep Duration

**Figure 2:**
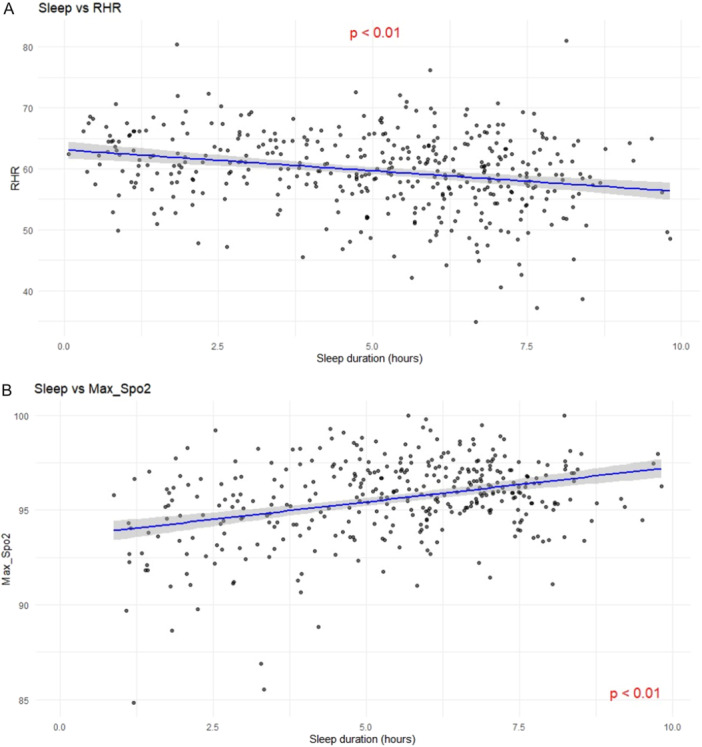
(A) Scatter plot demonstrating the relationship between sleep duration and resting heart rate (RHR). Longer sleep duration was associated with lower RHR (*p* < 0.01). (B) Scatter plot demonstrating the relationship between sleep duration and maximum oxygen saturation (MaxSpO₂). Longer sleep duration was associated with higher MaxSpO₂ (*p* < 0.01).

Short sleep duration was also associated with a lower MaxSpO₂. Individuals sleeping ≤ 6 h had a mean MaxSpO₂ of 95.1 ± 2.4%, compared with 96.2 ± 1.5% among those sleeping more than 6 h (mean difference −1.1%, *p* < 0.001) (Figure 2).

### 3.4 Continuous Sleep Duration and Outcomes

In multivariable linear regression analysis, shorter sleep duration was independently associated with higher RHR (*β* = 0.71, 95% CI 0.46–0.96; *p* < 0.001) and lower maximum oxygen saturation (*β* = −0.40, 95% CI − 0.51 to −0.29; *p* < 0.001). HRV, age, BMI, CHA₂DS₂‐VASc score, and sex were not significantly associated with the outcomes (Table 3).

**Table 3:** Multivariable linear regression analysis of clinical and smartwatch‐derived variables associated with sleep duration treated as a continuous variable.

Variable	β Coefficient	SE	95% CI	p‐value
HRV	−0.08	0.05	−0.17 to 0.02	0.14
RHR	0.71	0.13	0.46 to 0.96	**< 0.001**
Age	−0.45	0.25	−0.95 to 0.04	0.07
BMI	0.17	0.15	−0.11 to 0.46	0.23
CHA₂DS₂‐VASc	−0.03	0.02	−0.06 to 0.00	0.09
Max SpO₂	−0.4	0.06	−0.51 to −0.29	**< 0.001**
Gender	0.31	0.24	−0.16 to 0.77	0.20

*Note:* Bold value indicate important and significant outcomes.

### 3.5 Hypoxia and Short Sleep

Short sleep duration (≤ 6 h) was significantly associated with higher odds of nocturnal hypoxia burden with affected patients having 2.58‐fold higher odds compared with those sleeping > 6 h (95% CI 1.02–7.41; *p* = 0.044).

## 4 Discussion

In this study of 336 participants monitored with Samsung Galaxy Watches from the HEARTBEAT study, average nightly sleep duration less than 6 h was associated with higher RHR and lower maximum oxygen saturation (MaxSpO_2_) during sleep compared to patients with an average sleep duration more than 6 h. These differences remained significant after matching on baseline characteristics and comorbidities, including obstructive sleep apnea. This suggests that short sleep duration measured from wearables is linked to autonomic and respiratory alterations that may have cardiovascular relevance (Central illustration 1).

**Central Illustration 1:**
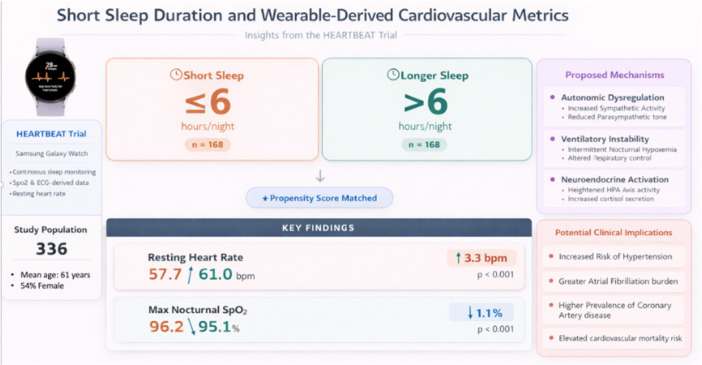
Short Sleep and Cardiovascular Metrics in the HEARTBEAT. Among 336 propensity score‐matched participants, average sleep duration ≤ 6 h/night was associated with higher resting heart rate (+3.3 bpm) and lower maximum nocturnal oxygen saturation (−1.1%) measured by Samsung Galaxy Watch (both *p* < 0.001).

### 4.1 Association Between Short Sleep Duration With RHR and SpO_2_

The observed increase in RHR of 3.3 beats per minute among the shorter sleep group is consistent with prior research demonstrating that sleep deprivation increases sympathetic nervous system activity and reduces parasympathetic modulation, resulting in elevated heart rates [2, 10]. Several studies have demonstrated that even modest reductions in sleep duration are associated with increased circulating catecholamines and cortisol activation, which contribute to higher heart rate and blood pressure [11, 12, 13, 14].

The reduction of 1.1% in nocturnal MaxSpO₂ in the short‐sleeping group also reflects underlying physiological changes, and insufficient sleep may alter respiratory physiology through mechanisms distinct from apnea‐related desaturation. Sleep deprivation has been shown to reduce both hypoxic and hypercapnic ventilatory responses by approximately 25–30%, which may contribute to subtle reductions in nocturnal oxygenation [15]. However, previous studies demonstrating this association in individuals without sleep‐disordered breathing are limited, and our finding of lower MaxSpO₂ in short sleepers independent of OSA status is novel.

### 4.2 Cardiovascular Significance of RHR and SpO_2_

The observed changes have important implications for patient outcomes. Growing evidence suggests that inadequate sleep contributes to impaired vascular function and elevated long‐term cardiovascular risk and mortality [16, 17]. Evidence from cardiac MRI showed that sleep deprivation among medical professionals led to increased cardiac contractility due to high sympathetic activity, which can further explain the elevated RHR [18]. Elevated RHR is a well‐established predictor of hypertension, coronary artery disease, atrial fibrillation, and all‐cause mortality. Fox et al. demonstrated that RHR is an independent predictor of cardiovascular and all‐cause mortality in men and women with and without diagnosed cardiovascular disease, and leads to the progression of coronary atherosclerosis, occurrence of myocardial ischemia, ventricular arrhythmias, and left ventricular dysfunction [5]. A comprehensive meta‐analysis of 46 prospective cohort studies involving over 1.2 million patients reported that each 10 beats/min increment in RHR was associated with approximately 10% increased risk of all‐cause mortality and cardiovascular mortality [19].

Similarly, reduced nocturnal oxygen saturation has been associated with endothelial dysfunction, oxidative stress, vascular inflammation, and higher long‐term cardiovascular risk [20, 21]. The recurrent periods of hypoxemia promote reactive oxygen species overproduction and increase inflammatory response, contributing to endothelial damage and atherogenesis [22]. Nocturnal oxygenation patterns, even in individuals without sleep apnea, have been linked to a higher risk of incident hypertension and metabolic disease. For instance, a reduced SpO₂, specifically maximal SpO₂, during the night has been associated with an increase in all‐cause mortality [6]. Thus, the combination of higher RHR and lower nocturnal oxygenation in short sleepers may represent a pathophysiologic pathway through which insufficient sleep contributes to elevated cardiovascular risk.

### 4.3 Clinical Implications of Wearable‐Derived Sleep Metrics

Integrating consumer wearables into sleep assessment has become increasingly important because these devices enable continuous, passive, real‐world monitoring of physiological signals that cannot be captured through traditional sleep questionnaires or single‐night polysomnography [12]. Furthermore, patients move less during sleep, making sleep measurements less prone to motion artifacts and therefore an important period for observation. Mekhael et al. demonstrated that consumer wearables can detect persistent alterations in sleep architecture among long‐COVID patients, highlighting their capability to identify deviations in sleep patterns in ambulatory populations [23].

A multicenter validation study evaluating 11 consumer sleep trackers, including major devices such as the Galaxy Watch 5, Apple Watch 8, Fitbit Sense 2, Google Pixel Watch, and Oura Ring, reported that widely used smartwatches can reliably monitor sleep compared with polysomnography [24].

Although the absolute differences in RHR and maximum nocturnal oxygen saturation observed in this study were modest, they remain important. Prior studies suggest that even small changes in physiologic parameters such as RHR and oxygen saturation may carry clinical significance over time [25]. Our findings suggest that wearables can identify subtle physiologic consequences of inadequate sleep, such as elevated heart rate and reduced blood oxygen saturation, and thus support their potential future role in monitoring physiologic trends and contributing to preventive health strategies.

## 5 Limitations

Several limitations should be acknowledged. First, the observational design of this study means these findings do not establish a direct causal relationship; however, extensive propensity‐score matching across multiple demographic and clinical variables was performed to mitigate confounding and strengthen the robustness of the associations. Second, nocturnal oxygen saturation was assessed using average maximum SpO₂ values rather than hypoxic burden metrics; future studies should incorporate cumulative measures of oxygen desaturation, which may better capture clinically relevant hypoxemia. Third, clinical outcome data were limited and therefore not included in the present analysis; future longitudinal studies integrating hard cardiovascular endpoints will be essential to further understand the clinical significance of our results. Fourth, sleep and physiological measurements were derived from a single wearable device and depended on participant adherence. Fifth, these measurements were derived from a single wearable platform, and results may not be generalizable to other devices with different hardware or proprietary algorithms. Sixth, given the observational design of this study, we were unable to evaluate whether interventions aimed at improving sleep would alter these physiologic parameters. Finally, although the Samsung Galaxy Watch has demonstrated reasonable accuracy, wearable‐derived metrics may be affected by device‐specific limitations, signal artifacts, and misclassification compared with gold‐standard polysomnography.

## 6 Conclusion

In participants monitored via Samsung Galaxy Watches, average nightly sleep duration of ≤ 6 h was associated with higher RHR and lower MaxSpO₂ during sleep. These findings underscore the importance of adequate sleep for cardiovascular and respiratory health. Our results highlight the potential value of integrating smartwatches into routine health monitoring and preventive strategies, as they may identify early cardiovascular and respiratory changes associated with insufficient sleep. Further studies are required to explore whether these findings are also linked to hard outcomes and whether improved sleep can ameliorate these physiological parameters and reduce long‐term cardiovascular risk.

## Ethics Statement

The Health Electronic Assessment of Risks and Trends Using Biometric Equipment and Technology (HEARTBEAT) study was approved by the Institutional Review Board at Tulane University (NCT06753045).

## Consent

Written informed consent was obtained from all participants prior to enrollment.

## Conflicts of Interest

NM reports having received consulting fees from Biosense Webster, Boston Scientific, and AtriCure. NM also reports being a speaker for Abbott, Biosense Webster, AtriCure, and Sanofi. NM also reports receiving research support from Abbott, Medtronic, Biosense Webster, Siemens, GE, Boston Scientific, Sanofi, and Samsung. NM also reports having a family member as the CEO of Cardiac Designs. NM also reports being the founder of Marrek, being named in a patent issued for MRI fibrosis imaging and being a previous shareholder of Cardiac Designs.

## Data Availability

The data supporting the findings of this study are available from the corresponding author upon reasonable request.
